# The association between the albumin to globulin ratio and thoracic spine bone mineral density in adolescents: NHANES 2011–2016

**DOI:** 10.3389/fnut.2025.1560013

**Published:** 2025-06-26

**Authors:** Haoyu Yan, Jie Yang, Yancheng Dai, Yan Li, Peng Zhang, Liuyan Li

**Affiliations:** ^1^Department of Orthopedics, The Second Affiliated Hospital of Soochow University, Suzhou, China; ^2^Department of Family Medicine, The Second Affiliated Hospital of Soochow University, Suzhou, China; ^3^Guangfu People’s Hospital, Suzhou, China

**Keywords:** albumin to globulin ratio (AGR), bone mineral density (BMD), osteoporosis, NHANES, adolescent

## Abstract

**Background:**

Numerous studies have demonstrated a strong correlation between osteoporosis and nutritional status and inflammation, while albumin and globulin are important references for nutritional status and inflammation, respectively. The albumin to globulin ratio (AGR), which combines the levels of albumin (ALB) and globulin (GLB), is a new comprehensive index that offers a more precise reflection of the inflammatory state of the body and its nutritional status. However, the connection between AGR and thoracic spine bone mineral density (BMD) remains poorly understood. The purpose of this research was to examine the link between the AGR and thoracic spine BMD in adolescents.

**Methods:**

This study used the most recent data from the National Health and Nutrition Examination Survey (NHANES) from 2011 to 2016, and used weighted multivariate linear regression modeling to examine the correlation between AGR and thoracic spine BMD in adolescents. Threshold effects and non-linear relationships were assessed using a smoothed curve-fitting algorithm alongside threshold effects analysis. Furthermore, subgroup analyses and interaction tests were performed.

**Results:**

The study comprised a total of 3,000 participants who were all aged 20 years or younger. Based on weighted multivariate linear regression analysis, in the fully adjusted model, a significantly higher thoracic spine BMD was found in the highest AGR quartile compared to the lowest AGR quartile (*p* < 0.05). After adjusting for variables, subgroup analyses showed no significant interaction effects. The study of threshold effects and the fitting of smooth curves identified a specific threshold effect for AGR and thoracic BMD with an inflection point of 1.237, after which AGR was significantly positively correlated with thoracic spine BMD.

**Conclusion:**

The study identified a notable positive correlation between AGR and adolescent thoracic spine BMD. This finding indicates a potential correlation between higher AGR and higher thoracic spine BMD, which may be indicative of a reduced risk of developing Osteoporosis (OP). This emphasizes the importance of considering nutritional and inflammatory status in the prevention of OP, thereby validating the utilization of AGR as a pivotal marker for the development of early intervention methodologies.

## Introduction

Osteoporosis (OP) is a systemic skeletal disorder distinguished by diminished bone mass and the breakdown of bone tissue microarchitecture. As a consequence, bone fragility is exacerbated, leading to a higher fracture risk (1, 2). Under the criteria established by the WHO (World Health Organization), osteoporosis affects an estimated 19.7% of the global population (3). Adolescence represents a pivotal period for the accrual of bone mineral, with substantial contributions to the attainment of peak bone mass. In osteological research, the loss of bone mass during adolescence is known to increase the risk of fractures in later life (4, 5). In conclusion, bone metabolism in adolescence exerts a significant influence on the development of osteoporosis in later life (6).

Recent research has increasingly emphasized the significant role of albumin (ALB) and globulin (GLB) in various clinical diseases. Albumin, the primary plasma protein produced by the liver, plays a critical role in regulating osmotic pressure, maintaining fluid balance, and transporting various substances such as hormones, drugs, and fatty acids. Additionally, it is commonly used as a biomarker to evaluate the body’s nutritional status (7). In contrast, globulin is closely associated with the inflammatory response, which is an important trigger for osteoporosis and disorders of bone metabolism (8, 9). In order to assess an individual’s nutritional and inflammatory status more comprehensively, the albumin-globulin ratio (AGR) was developed. This ratio offers a more precise assessment of the body’s inflammatory status and nutritional condition by simultaneously considering changes in both albumin and globulin levels (10). In recent years, AGR has gained widespread use in the study of various clinical diseases, including malignant tumors, acute ischaemic stroke and chronic heart failure (11–17). These studies suggest that changes in AGR may be closely linked to disease prognosis, clinical manifestations, and the overall health of the patient.

Drawing on data from the National Health and Nutrition Examination Survey (NHANES), this study sought to evaluate the correlation between AGR and thoracic spine bone mineral density (BMD) in adolescents aged 12–20 years in the United States.

## Methods

### Study population

**Figure 1:**
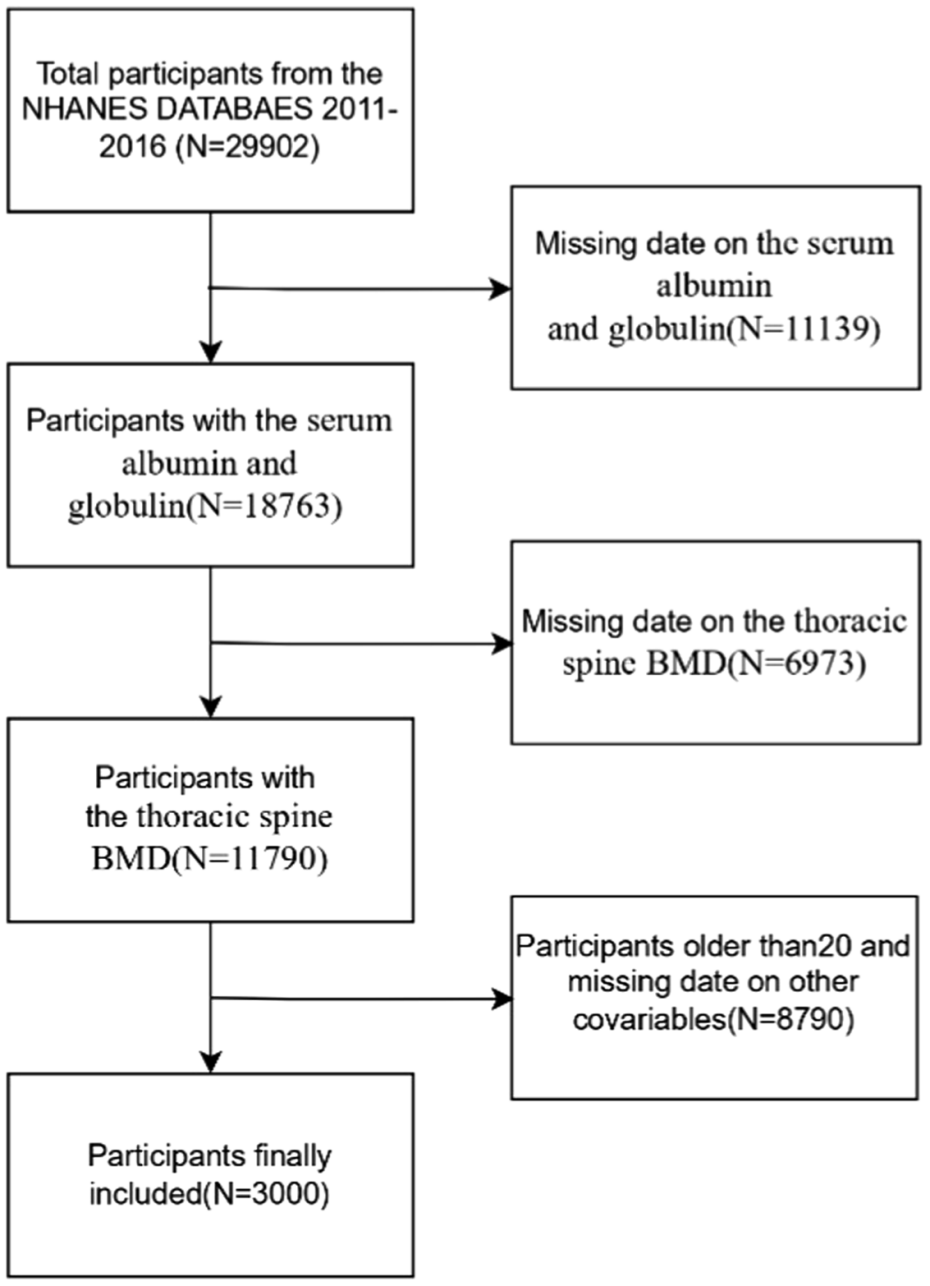
Study flowchart.

The data used in this study come from NHANES, an ongoing program, which is a national survey that measures the health and nutrition of U. S. population. The survey integrates interviews, clinical assessments, and laboratory analyses to provide comprehensive and reliable health-related data and is a valuable resource for population-based research. The NHANES employs complex sampling design weights to ensure national representativeness. Weights were calculated as the inverse of selection probabilities (base weights), adjusted for non-response, and calibrated via post-stratification to U. S. Census Bureau population benchmarks. For merged multi-cycle data, weights were adjusted proportionally (18). Further details can be found on the official website at https://www.cdc.gov/nchs/nhanes/index.htm. This study uses data from surveys conducted between 2011 and 2016, involving a total of 29,902 participants, and intends to assess the nutritional and health conditions of the U. S. population. Exclusion criteria were applied to remove participants with missing data on albumin and globulin (*n* = 11,139), thoracic spine BMD (*n* = 6,973), those older than 20 years (*n* = 8,466), and individuals with incomplete data on other relevant covariates, including BMI (*n* = 5), PIR (poverty-to-income ratio) (*n* = 283), total calcium (*n* = 13), hypertension (*n* = 1), diabetes (*n* = 21), and triglycerides (*n* = 1). For missing data, we imputed continuous variables using medians or means based on the data distribution and categorical variables using modes. After applying the exclusion criteria, the final sample consisted of 3,000 participants (Figure 1).

### Exposure variable

In the present study, AGR was designed as the exposure variable, which is the calculated ratio of serum albumin to serum globulin. The AGR is a comprehensive metric that evaluates both albumin and globulin levels and provides a more precise assessment of the body’s nutritional and inflammatory conditions.

### Outcome variable

The primary outcome indicator in this study was thoracic spine bone mineral density. Dual-energy X-ray absorptiometry (DXA) has been clinically validated for its safety and is suitable for use in all age groups (including children and the geriatric population). The young and healthy population demonstrate optimal accuracy (coefficient of variation ≤1%) in the measurement of spine and whole-body bone mass (19, 20). Consequently, DXA was utilized to assess bone mineral density in this study.

### Covariable

Based on previous studies, other relevant potential confounders were included, including demographic, questionnaire, screening, and laboratory data. The demographic data set includes variables such as age, gender, race, and the poverty-to-income ratio (PIR); questionnaires included diabetes (yes/no), hypertension (yes/no), smoking (yes/no), drinking (yes/no) and Physical activity (yes/no)(whether they met the American Physical activity guideline (at moderate intensity should be done for 150 min a week or, at vigorous intensity, should be performed 75 min/week for adults); screening data included body mass index (BMI) and thoracic spine BMD; and laboratory data included alkaline phosphatase (ALP), serum calcium (Ca), phosphorus (P), Alanine transaminase (ALT), Aspartate aminotransferase (AST), blood urea nitrogen (BUN), albumin (ALB), globulin (GLB), total protein, total protein and triglycerides (TG). Details of how these variables are calculated can be found on the NHANES website.

### Statistical analysis

For continuous variables, the mean ± standard deviation (mean ± SD) was used, while categorical variables are reported as percentages. In order to facilitate meaningful comparisons, the continuous variables were analyzed using weighted *t*-tests, while the categorical variables were analyzed using chi-square tests. The results obtained from this analysis are presented as counts (n) and percentages (%). The correlation between AGR and thoracic spine BMD was examined using weighted multiple linear regression analysis across three different models, with the lower quartile serving as the reference group. Model 1 served as the baseline, with no adjustments for any variables; Model 2 adjusted for age, gender, and race; and Model 3 incorporated a broader range of factors, including gender, age, race, PIR, ALP, Ca, P, ALT, AST, BUN, total protein, TG, diabetes, hypertension, smoking, drinking, and BMI. Subsequently, an assessment was conducted to determine the existence of a non-linear relationship between AGR and thoracic spine BMD. This assessment employed a smoothed curve-fitting technique in conjunction with a threshold effects assessment. Subsequent to this, subgroup analyses were performed in the ultimate stage of data analysis. EmpowerStats was employed for the statistical analysis in this study (accessible at http://www.empowerstats.com). Statistical significance was defined as *p* < 0.05.

## Results

### Study population features

The study included 3,000 participants, with a mean age of 15.69 ± 2.45 years. Of the cohort, 46.9% were female and 53.1% were male, with an average AGR of 1.65 ± 0.30. Among the different groups AGR (quartiles, Q1–Q4), all covariates were statistically significant (*p* < 0.05), except for hypertension status, which did not achieve statistical significance (*p* > 0.05) (Table 1).

**Table 1:** Weighted sample characteristics for the study.

	Q1 (*N* = 750) 0.540–1.45	Q2 (*N* = 718) 1.45–1.63	Q3 (*N* = 768) 1.63–1.81	Q4 (*N* = 764) 1.81–8.17	*p*-value
Gender (%)					<0.001
Male	34.345	42.752	57.700	70.127	
Female	65.655	57.248	42.300	29.873	
Age (years)	15.995 ± 2.561	16.074 ± 2.721	15.921 ± 2.511	15.705 ± 2.478	0.027
Race (%)					<0.001
Mexican American	15.406	15.872	13.248	11.853	
Non-Hispanic Black	8.564	8.803	7.366	5.444	
Non-Hispanic White	38.042	49.639	62.547	69.84	
Other Hispanic	27.188	16.501	9.437	5.350	
Other Race	10.800	9.185	7.402	7.512	
PIR	2.235 ± 1.592	2.244 ± 1.587	2.438 ± 1.606	2.623 ± 1.618	<0.001
ALT (U/L)	20.889 ± 15.962	21.742 ± 19.845	19.595 ± 10.205	18.898 ± 10.494	<0.001
AST (U/L)	23.350 ± 7.642	24.909 ± 15.782	24.511 ± 13.359	23.516 ± 6.854	0.025
ALP (IU/L)	114.877 ± 78.649	116.707 ± 83.676	126.092 ± 87.179	142.342 ± 100.332	<0.001
Total calcium (mg/dL)	9.511 ± 0.312	9.570 ± 0.286	9.655 ± 0.296	9.665 ± 0.287	<0.001
Blood urea nitrogen (mmol/L)	10.416 ± 3.875	10.926 ± 3.087	11.185 ± 3.300	11.472 ± 3.299	<0.001
Phosphorus (mg/dL)	4.232 ± 0.676	4.294 ± 0.670	4.299 ± 0.682	4.350 ± 0.678	0.011
Total protein (g/dL)	7.465 ± 0.414	7.284 ± 0.367	7.241 ± 0.379	7.035 ± 0.373	<0.001
Triglycerides (mg/dL)	108.099 ± 75.467	101.714 ± 74.957	100.836 ± 72.729	92.031 ± 57.030	<0.001
Albumin (g/L)	42.399 ± 2.836	44.200 ± 2.313	45.743 ± 2.382	46.956 ± 2.470	<0.001
Globulin (g/L)	32.249 ± 2.902	28.641 ± 1.516	26.669 ± 1.549	23.386 ± 2.019	<0.001
BMI (kg/cm^2^)					<0.001
<25	48.695	57.755	66.795	76.053	
> = 25	51.305	42.245	33.205	23.947	
Smoking (%)					0.039
Yes	4.561	4.053	7.222	5.700	
No	95.439	95.947	92.778	94.300	
Drinking (%)					<0.001
Yes	84.510	85.826	90.691	92.939	
No	15.490	14.174	9.309	7.061	
Hypertension (%)					0.295
Yes	2.576	1.247	1.801	1.503	
No	97.424	98.753	98.199	98.497	
Diabetes (%)					0.011
Yes	1.044	0.536	0.149	0.050	
No	98.956	99.464	99.851	99.950	
Physical activity					<0.001
Yes	20.944	22.719	33.024	24.853	
No	79.056	77.281	66.976	75.147	
Thoracic spine BMD (g/cm^2^)	0.759 ± 0.112	0.754 ± 0.106	0.743 ± 0.104	0.737 ± 0.110	<0.001

For continuous variables, the mean ± standard deviation (mean ± SD) was used, *p*-value was calculated by weighted linear regression model. While categorical variables are reported as percentages, *P*-value was calculated by weighted chi-square test.

### Connection of AGR with thoracic spine BMD

To examine the correlation between AGR and thoracic spine BMD, a weighted multivariate linear regression model was used in this study (Table 2). In Model 1, without adjustment for any confounders, a significant negative correlation was observed between AGR and thoracic spine BMD (*β* = −0.002, 95%CI: −0.033, −0.011, *P* < 0.001). In model 2, after adjustment for age, gender, and race, there was no significant correlation between AGR and thoracic spine BMD (*β* = −0.007, 95% CI: −0.017, 0.003, *p* = 0.084). In Model 3, after adjustment for all potential confounders, a significant positive correlation was found between AGR and thoracic spine BMD (*β* = 0.019, 95%CI: 0.007, 0.030, *p* = 0.002). Specifically, in Model 3, thoracic spine BMD was 0.019 g/cm^2^ higher in the highest quartile compared to the lowest quartile.

**Table 2:** Association between AGR and thoracic spine BMD.

Exposure	Model 1 β (95% CI), *p*-value	Model 2 β (95% CI), *p*-value	Model 3 β (95% CI), *p*-value
AGR	−0.030 (−0.044, −0.017) < 0.001	−0.016 (−0.028, −0.004) 0.017	−0.016 (−0.030, −0.002) 0.029
AGR quartile			
Q1	Reference	Reference	Reference
Q2	−0.004 (−0.016, 0.008) 0.478	0.001 (−0.010, 0.011) 0.902	0.011 (0.001, 0.021) 0.043
Q3	−0.016 (−0.027, −0.005) 0.006	−0.007 (−0.017, 0.003) 0.151	0.010 (−0.001, 0.020) 0.048
Q4	−0.022 (−0.033, −0.011) <0.001	−0.007 (−0.017, 0.003) 0.163	0.019 (0.008, 0.031) 0.001
*P* for trend	<0.001	0.084	0.002

Model 1 was unadjusted; Model 2 was adjusted for gender, age, and race; and Model 3 further adjusted for PIR, ALP, Ca, P, ALT, AST, BUN, total protein, TG, diabetes, hypertension, smoking, drinking status, BMI and Physical activity.

### Non-linear relationship between AGR and thoracic spine BMD

**Figure 2:**
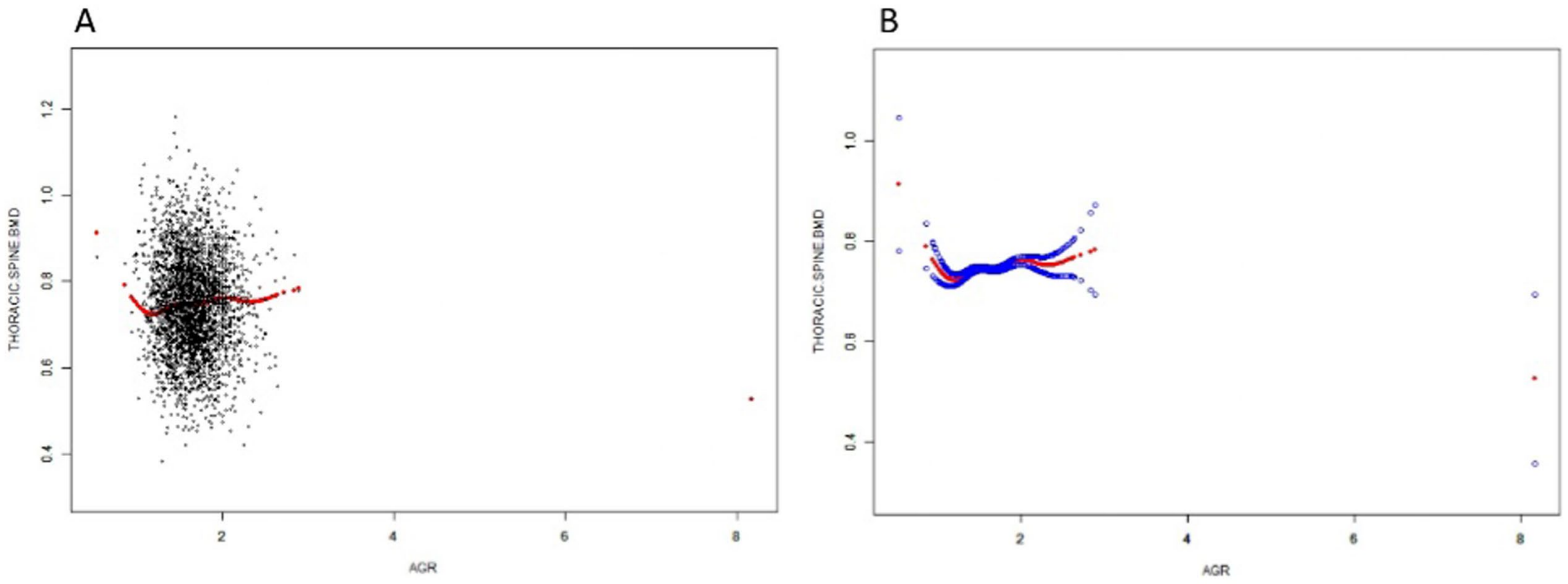
The Relationship between AGR and Thoracic spine BMD. **(A)** Each black dot on the graph denotes a single sample and the red line represents the fitted line for all participants. **(B)** The figure shows a smooth curve fit between the variables indicated by the red line. The blue line shows the 95% confidence interval of the fit.

In this study, we used a smoothed curve-fitting technique to further investigate the correlation between AGR and thoracic spine BMD. Figure 2 shows a complex, non-linear U-shaped association between AGR and thoracic spine BMD. To explore this correlation further, a threshold effect analysis was performed using a weighted two-segment linear regression model, with all confounding factors fully adjusted for. An inflection point of 1.237 for AGR was identified in this analysis, with the likelihood ratio test showing a statistically significant *p*-value of <0.001. Below the AGR threshold of 1.237, each 1-unit increase in AGR was associated with a decrease in thoracic spine BMD of 0.213 g/cm^2^ (*β* = −0.213, 95%CI: −0.311, −0.114). Above this threshold, a one-unit increase in AGR was linked to a 0.018 g/cm^2^ rise in thoracic spine BMD (*β* = 0.018, 95% CI: 0.004, 0.032) (Table 3).

**Table 3:** Threshold effect analysis of AGR and thoracic spine BMD.

Thoracic spine BMD	β (95% CI)	*p*-value
Model I	0.011 (−0.003, 0.025)	0.1180
Model II		
Inflection point (K)	1.237	
<K point effect 1	−0.211 (−0.310, −0.113)	<0.0001
>K point effect 2	0.018 (0.004, 0.033)	0.0112
Effect 2 minus effect	0.230 (0.129, 0.330)	<0.0001
Predicted value of the equation at the folding point	0.757 (0.750, 0.765)	
Log-likelihood ratio test		<0.001

Adjusted for gender, age, race, PIR, ALP, total calcium, P, ALT, AST, BUN, total protein, TG, diabetes, hypertension, smoking, drinking status, BMI and Physical activity.

Model I: univariate linear regression.

Model II: two-part regression model.

### Subgroup analysis

**Figure 3:**
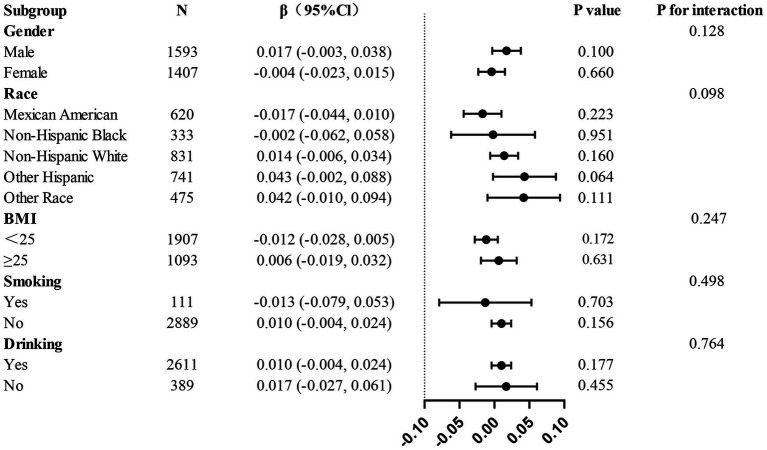
Subgroup analysis of the associations between AGR and thoracic Spine BMD.

The present study conducted a subgroup analysis in order to examine the relationship between AGR and thoracic spine BMD across various populations. When participants were grouped by age, gender, race, smoking, drinking, hypertension, and diabetes status, no statistically significant interactions were detected across any of the subgroups (Figure 3), further validating the consistency of the role of AGR in adolescents aged 12–20 years.

## Discussion

The study sought to evaluate the relationship between AGR and thoracic spine BMD in a sample of adolescents aged 12–20 years residing in the U. S. A cross-sectional study involving 3,000 participants demonstrated a positive correlation between AGR and thoracic spine BMD in a fully adjusted model of weighted multiple linear regression. Curve fitting and threshold effect analyses revealed a non-linear relationship with an inflection point of 1.237. When AGR ≤ 1.237, thoracic spine BMD decreased with increasing AGR. When AGR ≥ 1.237, thoracic spine BMD increased with increasing AGR. Subgroup analyses and interactions showed no significant interactions between the different subgroups.

Earlier research has concentrated on the impact of albumin or globulin on BMD, and this research is the first to explore the connection between the AGR and thoracic spine BMD, establishing it as a pioneering study in the field. This approach integrates nutritional and inflammatory biomarkers to provide a more nuanced understanding of bone health. As demonstrated in numerous preceding studies, albumin plays a pivotal role in maintaining optimal bone health, functioning as an indicator of nutritional status. A large-scale U. S. study demonstrated a notable correlation between lower serum albumin levels and reduced BMD, with the duration of hypoalbuminemia strongly correlating with the severity of BMD deterioration (21). Another study similarly demonstrated that individuals with hypoalbuminemia faced a notably increased risk of developing osteoporosis in comparison with individuals with normal albumin levels (22, 23). In addition, several studies have indicated that low albumin levels may elevate the risk of fracture (22, 24). In contrast, another study found no association between serum albumin and osteoporosis (25). Serum globulin contains a variety of proteins involved in the inflammatory response, such as complement, immunoglobulins, and acute-phase reactive proteins, and have been recognized as reliable indicators of inflammatory status (26, 27). Specifically, serum globulin include: immunoglobulins, proteins of the complement system, and acute phase response proteins [e.g., C-reactive protein (CRP), ferritin, and α1-antitrypsin]. A number of studies have indicated that immunoglobulins and complement proteins can exacerbate the onset and progression of osteoporosis by contributing to the inflammatory response and promoting oxidative stress. These factors can subsequently accelerate the onset and progression of osteoporosis through multiple mechanisms, including the promotion of osteoclast activation, the inhibition of osteoclast function, and the alteration of the skeletal microenvironment (28). Several studies have shown that elevated levels of C-reactive protein (CRP) are linked to a reduction in BMD (29, 30); and some studies have found that elevated serum ferritin levels may indicate an increased risk of bone loss (31–33). In contrast to single indicators, AGR takes into account the combined effects of albumin and globulin on BMD, which may reflect not only changes in nutritional status but also inflammatory status in the mid-to long-term (12, 34).

The present study observed a U-shaped relationship between AGR and thoracic spine BMD, which began to decrease as AGR increased and then decreased after reaching the inflection point of AGR 1.237 levels, which reflect nutritional status, decrease during inflammation, while globulin levels increase (35). Total serum protein is predominantly constituted of albumin (approximately 50%) and globulin (approximately 48%). Albumin, a pivotal nutritional assessment index and a negative acute time-phase response protein, exhibits changes in its concentration. These changes can synchronize the nutritional and metabolic status of the body and the degree of inflammatory response. Abnormally low levels of this protein have been observed in a range of clinical conditions, including malnutrition syndromes, hepatic and renal dysfunction, and systemic inflammatory diseases (36). Globulin fractions are primarily constituted of immunoglobulin family and acute phase response proteins, and their biosynthesis is modulated by pro-inflammatory cytokines such as IL-6 and TNF-*α*. In the context of chronic inflammatory pathologies, these proteins exhibit a characteristic increase in concentration, and their dynamic changes can provide a valuable reference point for the clinical assessment of infectious diseases and chronic inflammatory states (37). In cases where the AGR is low, this may be indicative of malnutrition or chronic disease, as lower albumin levels are commonly associated with a poorer nutritional status or inadequate protein synthesis. It has been hypothesized that low albumin levels may result in bone loss. This is due to the fact that inadequate protein synthesis may interfere with the formation of bone matrix. Furthermore, lower AGR has been associated with pathologies such as chronic inflammation and abnormal liver function, which themselves can negatively affect bone metabolism and lead to decreased bone density. As the AGR increases, it is indicative of a gradual improvement in the albumin to globulin ratio, which may reflect improved nutritional status or reduced levels of chronic inflammation. In conclusion, the compensatory increase of globulin, stimulated by inflammatory factors (IL-6, TNF-*α*, etc.) and the consumptive decrease of albumin, as a negative acute time-phase reactive protein form, establish a dynamic equilibrium. This characteristic inverse fluctuation pattern significantly enhances the specificity of the AGR for the identification of inflammatory versus infectious states. An increase in AGR value is suggestive of the body’s gradual realization of homeostatic reconstruction of inflammation regulation and nutrient metabolism. The restoration of this physiological balance has the capacity to promote the maintenance of a normal bone metabolism microenvironment and the homeostasis of bone density. It is evident that the AGR is of significant clinical value in the evaluation of inflammation-related diseases.

The relationship between albumin levels and BMD and osteoporosis can be explained in several ways. Firstly, albumin has antioxidant capacity (38), and low albumin levels reduce its antioxidant effect, leading to enhanced oxidative stress, which in turn triggers mitochondrial dysfunction and DNA damage (39, 40), contributes to bone marrow mesenchymal stem cell senescence (41, 42), affects osteogenesis, and increases the risk of osteoporosis; second, low albumin levels may promote osteoclasts through activation of NF-κB pathway differentiation and survival (43, 44), leading to osteolysis and further exacerbating osteoporosis. Finally, low albumin levels may affect the metabolism of bone formation-related proteins and reduce the formation of calcium phosphate apatite crystals, thereby decreasing BMD and increasing fracture risk (45). Globulin, on the other hand, act more through inflammatory states. In chronic inflammatory states, globulin levels rise, and the production of inflammatory mediators such as tumor necrosis factor (TNF-*α*) and interleukin-6 (IL-6) is heightened. TNF-α stimulates osteoclast differentiation, promotes the expression of RANK in osteoclast precursors, and facilitates RANKL-induced osteoclastogenesis (46–48). IL-6 activates the JAK-STAT3 signaling pathway, which enhances the production of RANKL by stromal cells and osteoblasts. This increase in RANKL stimulates osteoclastogenesis, promoting osteoclast activation (49–51). All of these ultimately promote osteoclast activation, leading to increased bone resorption and decreased BMD.

### Limitation and strengths

Several limitations should be considered in this study. Firstly, the cross-sectional design restricts the ability to determine a causal correlation between AGR and thoracic spine BMD. In order to corroborate these findings and elucidate the underlying mechanisms, it is imperative that longitudinal studies are conducted. Second, the study focused only on individuals aged ≤20 years, limiting the applicability of the results to other populations, such as the elderly, who may exhibit different AGR-BMD dynamics. Third, although full adjustment was made for known confounders, it was not possible to exclude the potential influence of unmeasured variables such as genetic factors, physical activity levels, or specific dietary patterns. Fourth, direct indicators of pubertal development (e.g., Tanner staging or bone age) were not systematically recorded in the NHANES data, although we performed sensitivity analyses stratified by age and sex, and these alternative methods may not fully capture individual differences in biological maturity. Finally, as an observational study, it did not explore the biological mechanisms between AGR and BMD, emphasizing the need for experimental studies to better understand these pathways. Despite these limitations, this study has significant strengths. It benefited from a large, diverse, and well-characterized sample, increasing the reliability and robustness of the findings. The analysis included rigorous adjustment for numerous potential confounders to ensure the validity of the observed correlations. Non-linear relationships and saturation effects between AGR and BMD were identified using advanced statistical methods like Smooth curve fitting and threshold effects analysis, offering a more detailed understanding of their connection. In addition, the novel focus of this study on AGR as a combined biomarker of nutritional and inflammatory status provides new insights into the factors that influence bone health. Importantly, these findings highlight the potential of AGR as an early marker for identifying individuals at risk of impaired bone health, especially during adolescence, a critical period for bone development and osteoporosis prevention.

## Conclusion

The present study demonstrated a notable positive correlation between AGR and thoracic spine BMD in adolescents, thereby identifying a complex non-linear association between the two. Notably, when AGR is lower than 1.237, thoracic spine BMD decreased with increasing AGR. This study provides a new perspective on the clinical use of AGR as a predictor of BMD, especially in adolescent and young individuals, where lower AGR may indicate potential bone health risks. Changes in AGR may signal trends in BMD changes. Consequently, monitoring AGR in a clinical context may facilitate the early detection of bone quality issues and intervention.

## Data Availability

The datasets presented in this study can be found in online repositories. The names of the repository/repositories and accession number (s) can be found: https://wwwn.cdc.gov/Nchs/Nhanes/.
